# A specific synbiotic-containing amino acid-based formula restores gut microbiota in non-IgE mediated cow’s milk allergic infants: a randomized controlled trial

**DOI:** 10.1186/s13601-019-0267-6

**Published:** 2019-05-31

**Authors:** Harm Wopereis, Marleen T. J. van Ampting, Aysun Cetinyurek-Yavuz, Rob Slump, David C. A. Candy, Assad M. Butt, Diego G. Peroni, Yvan Vandenplas, Adam T. Fox, Neil Shah, Guus Roeselers, Lucien F. Harthoorn, Louise J. Michaelis, Jan Knol, Christina E. West, the ASSIGN study group

**Affiliations:** 1 Danone Nutricia Research, Uppsalalaan 12, 3584 CT Utrecht, The Netherlands; 2 Laboratory of Microbiology, Wageningen University, Stippeneng 4, 6708 WE Wageningen, The Netherlands; 3 Royal Alexandra Children’s Hospital, Brighton, UK; 4 University Hospital Verona, Verona, Italy; 5 Present Address: Department of Clinical and Experimental Medicine, University of Pisa, Pisa, Italy; 6 KidZ Health Castle, UZ Brussel, Vrije Universiteit Brussel, Brussels, Belgium; 7 Guy’s and St Thomas’ Hospitals NHS Foundation Trust, London, UK; 8 Great Ormond Street Hospital, London, UK; 9 Great North Children’s Hospital, Royal Victoria Infirmary, Newcastle upon Tyne, UK; 10 Umeå University, Umeå, Sweden

**Keywords:** Cow’s milk allergy, Pediatrics, Gut microbiota, Prebiotics, Probiotics

## Abstract

**Background:**

Altered gut microbiota is implicated in cow’s milk allergy (CMA) and differs markedly from healthy, breastfed infants. Infants who suffer from severe CMA often rely on cow’s milk protein avoidance and, when breastfeeding is not possible, on specialised infant formulas such as amino-acid based formulas (AAF). Herein, we report the effects of an AAF including specific synbiotics on oral and gastrointestinal microbiota of infants with non-IgE mediated CMA with reference to healthy, breastfed infants.

**Methods:**

In this prospective, randomized, double-blind controlled study, infants with suspected non-IgE mediated CMA received test or control formula. Test formula was AAF with synbiotics (prebiotic fructo-oligosaccharides and probiotic *Bifidobacterium breve* M-16V). Control formula was AAF without synbiotics. Healthy, breastfed infants were used as a separate reference group (HBR). Bacterial compositions of faecal and salivary samples were analysed by 16S rRNA-gene sequencing. Faecal analysis was complemented with the analysis of pH, short-chain fatty acids (SCFAs) and lactic acids.

**Results:**

The trial included 35 test subjects, 36 controls, and 51 HBR. The 16S rRNA-gene sequencing revealed moderate effects of test formula on oral microbiota. In contrast, the gut microbiota was substantially affected across time comparing test with control. In both groups bacterial diversity increased over time but was characterised by a more gradual increment in test compared to control. Compositionally this reflected an enhancement of *Bifidobacterium* spp. and *Veillonella* sp. in the test group. In contrast, the control-fed infants showed increased abundance of adult-like species, mainly within the *Lachnospiraceae* family, as well as within the *Ruminococcus* and *Alistipes* genus. The effects on *Bifidobacterium* spp. and *Lachnospiraceae* spp. were previously confirmed through enumeration by fluorescent in situ hybridization and were shown for test to approximate the proportions observed in the HBR. Additionally, microbial activity was affected as evidenced by an increase of l-lactate, a decrease of valerate, and reduced concentrations of branched-chain SCFAs in test versus control.

**Conclusions:**

The AAF including specific synbiotics effectively modulates the gut microbiota and its metabolic activity in non-IgE mediated CMA infants bringing it close to a healthy breastfed profile.

*Trial registration* Registered on 1 May 2013 with Netherlands Trial Register Number NTR3979.

## Background

The prevalence of food allergy in infancy and childhood is increasing in many countries worldwide. Cow’s milk allergy (CMA) is among the most common food allergies in early life and is associated with growth retardation throughout childhood, particularly in children suffering from persistent milk allergy [1]. Comorbidity is common, and many children develop other allergic conditions over time, also referred to as the allergic March [2]. The microbes that colonize the mucosal tissues after birth have a pivotal role in both innate and adaptive immune development [3, 4] and may have long-term effects both on the susceptibility and the persistence of allergic disease [5, 6].

Breastfeeding provides the infant gastrointestinal tract with a plethora of bioactive factors and has profound effects on gut microbiota composition and functions [7–9] and, as more recently reported, on oral microbiota development [10, 11]. Infants who suffer from severe CMA rely on cow’s milk protein avoidance and, when breastfeeding is not possible, require specialised infant formulas such as extensively hydrolysed formula (eHF) or amino acid-based formula (AAF) [12]. Incorporation of prebiotics, probiotics, or their combination (synbiotics) in these formulas offers a safe, suitable and effective strategy for both the dietary management and for potentially optimizing microbiota development in both IgE- and non-IgE-mediated CMA infants [13, 14].

In a randomized controlled trial with non-IgE-mediated CMA infants (ASSIGN study), an improvement of gut microbiota was observed in infants receiving an AAF with specific synbiotics (test) compared to infants receiving the same AAF without synbiotics (control). This improvement was based on an enhancement of bifidobacteria and a decrease of the *Eubacterium rectale/Clostridium coccoides* (ER/CC) group; in both test levels were close to the levels observed for a separate healthy, breastfed reference (HBR) group after an 8-week intervention [15]. Recently, it was reported that the levels of both microbial groups were sustained for the full study period of 26 weeks [16]. The fluorescence in situ hybridization (FISH) method used is an effective approach to quantify specific bacterial groups, but it does not provide information on the full bacterial composition and diversity of the community. For this, application of next-generation sequencing technologies are typically needed [17, 18]. We hypothesized that synbiotics would have a more comprehensive effect on the microbiota composition and activity. Therefore, we performed an in-depth characterisation of the microbial compositions of both faecal and saliva specimens collected in the ASSIGN study through 16S ribosomal RNA (16S rRNA)-gene sequencing, and in addition investigated the effects on gut physiology and bacterial metabolic activity by analysis of faecal pH, short-chain fatty acids (SCFA) and lactate.

## Methods

### Study design

ASSIGN was a prospective, randomized, double-blind controlled study (Netherlands Trial Register NTR3979) including infants with suspected non-IgE mediated CMA and a separate non-randomized healthy, breastfed reference group (HBR). Detailed methods on how the trial was conducted, and the primary and secondary outcome measures, have been published previously [15, 16].

In brief, subjects < 13 months of age with non-IgE mediated CMA were stratified based on predominant, investigator-assessed symptoms (skin or gastrointestinal) and randomly allocated to receive test (n = 35) or control formula (n = 36). Study duration was 26 weeks with allocation to study product for at least 8 weeks. After 8 weeks, randomized subjects continued to use the allocated study product or switched to commercially available eHF, or other milk substitute as per clinical practice guidelines of each medical centre. Subjects in the HBR group were age-matched to week 8 of the randomized groups (n = 51). Infants in the test group received an AAF (Neocate LCP; Nutricia Advanced Medical Nutrition, Liverpool, UK) including a prebiotic blend of chicory-derived neutral oligofructose and long-chain inulin (BENEO-Orafti SA, Oreye, Belgium) (9:1 ratio at a total concentration of 0.63 g/100 ml) and a probiotic strain *Bifidobacterium breve* M-16V (Morinaga Milk, Tokyo, Japan) at a concentration of 1.47 × 10^9^ colony-forming units (CFU)/100 ml formula. The control formula was a commercially available AAF (Neocate LCP; Nutricia Advanced Medical Nutrition, Liverpool, UK).

### Collection of saliva and stool samples

Saliva samples were collected from randomized infants at baseline and 8 weeks by a healthcare professional using the SalivaBio Children’s Swab method (Salimetrics, Carlsbad, USA) at least 1 h after feeding. Stool samples from randomized infants were collected, as reported previously [15], by parents/guardians at baseline, 8, 12 and 26 weeks. Parents/guardians of the age-matched non-randomized infants in the HBR group were asked to collect stool samples only. Saliva and stool specimens collected in the clinic were immediately frozen at − 80 °C. Stool specimens collected at home were immediately frozen in home-freezers and transported with ice-packs to the clinic by parents/guardians or by courier for storage at − 80 °C. Thereafter, both saliva and stools were transported on dry-ice (solid CO_2_) to Nutricia Research and stored at − 80 °C until analysis.

### DNA extraction

DNA extraction from saliva samples was performed with DNeasy Blood and Tissue Kits (Qiagen, Venlo, the Netherlands) according to the manufacturer’s protocol, except for an adaptation in the enzymatic lysis step and the addition of a mechanical lysis step as pre-treatment before the DNA isolation procedure. In brief, 150 µl of saliva sample was diluted up to 350 µl in PBS buffer (150 mM NaCl, 10 mM Na2HPO4, 20 mM NaH2PO4, pH 7.4) to which 50 µl of lytic enzymatic cocktail was added (50 mg/ml lysozyme, Sigma-Aldrich, St. Louis, Missouri, United States, USA and 20 µl proteinase K from Qiagen kit) and 300 mg of 0.1 mm glass beads (Biospec, Bartlesville, Oklahoma, USA). This suspension was incubated at 37 °C for 30 min, followed by one round of bead-beating for 10 min at 25 Hz (Tissuelyser I, Qiagen, Venlo, the Netherlands) and followed by the QIAcube isolation procedure (Qiagen).

DNA extraction from stools samples was performed with QIAmp DNA Stool Mini Kit (Qiagen, Venlo, the Netherlands) according to the manufacturer’s protocol except for the addition of two bead-beating steps as described before [19]. Extracted DNA from stools were purified from extraction impurities using spin columns (DCC™, Zymo research, Irvine, California, USA).

### Microbiota profiling

Faecal and salivary microbiota compositions were profiled by sequencing the hypervariable V3–V4 regions of the 16S rRNA gene. Sequencing was performed by LifeSequencing S.L. (Valencia, Spain) on an Illumina MiSeq instrument (San Diego, California, USA). The V3–V4 region was PCR-amplified with universal primers S-D-Bact-0341-b-S-17 primer (forward 5′-CCTACGGGNGGCWGCAG-3′) and S-D-Bact-0785-a-A-21 primer (reverse 5′-GACTACHVGGGTATCTAATCC-3′) [20] designed for dual indexing following the Illumina 16S Metagenomic Sequencing Library Preparation protocol (Part # 15044223 Rev. B). In brief, PCR amplification was performed in two steps: (1) in a first step, the V3–V4 region was amplified with the addition of universal adaptors to the amplification products. All amplicons were purified (AMPure XP, Beckman, Danvers, MA) to remove short amplification products and quantified using the Quant-iT PicoGreen dsDNA kit (Invitrogen, Carlsbad, California, USA). (2) In the second PCR step, the amplicons from the first step were amplified by targeting the universal adapters and with the addition of sample specific indexes and sequencing adaptors. The final amplicons were purified (AMPure XP) and quantified using the Quant-iT PicoGreen ds DNA kit (Invitrogen). All samples were pooled in equal amounts and sequenced in a 300 bp paired-end mode.

### Bioinformatic analysis of sequence data

Illumina reads were trimmed (removal of primers) and quality filtered by removing all reads with a mean q-score lower than 20 with ‘cutadapt v1.4.1’ [21]. Paired-end reads were merged using the program ‘PEAR v0.9.6’ [22]. Merged reads with q > 15 over a window of 5 bases, no ambiguous bases and a minimal length of 300 were retained and analysed with the ‘Quantitative Insights Into Microbial Ecology’ (QIIME) v1.9.0 package [23]. Sequences were clustered into operational taxonomic units (OTUs) based on 97% sequence identity as proxy for bacterial species using VSEARCH v2.03 with exclusion of chimeric sequences identified against the RDP gold database [24, 25]. Taxonomic assignment was performed using the RDP classifier [26] against the SILVA119 database [27]. Singleton OTUs, and OTUs with eukaryotic assignments, as well as OTUs with a low relative abundance (counts of an OTU as proportion of total reads of a sample) up to 0.005% were excluded from further downstream analysis. Representative sequences of OTUs were aligned using PyNAST [28] and used to build a phylogenetic tree with FastTree [29]. Rarefaction of the OTU tables was applied to account for the differences in sequencing depths (number of reads per sample) between the samples with default settings (10 equal depths from 10 sequences/sample up to the median number of sequences/sample with 10 iterations at each sequencing depth). The tree and rarefied OTU tables were used to calculate the species diversity (α-diversity) of the samples using Faith’s phylogenetic diversity (PD) [30] and the Shannon index for diversity [31].

The sequences within an OTU of interest (i.e. identified as differentially abundant from the statistical comparisons performed) were further partitioned into homogenous nodes with high sequence identity using the MED v2.1 algorithm [32]. Taxonomic assignment of the MED nodes were performed using the RDP classifier [26] against the SILVA119 database [27]. The assignment of the node with the largest number of reads and highest sequence identity was subsequently used as a more accurate proxy to the taxonomy of that OTU.

### Additional faecal sample parameters

To assess overall bacterial metabolic activity, the following faecal sample parameters were measured as described previously [33]: pH, concentrations of short-chain fatty acids (SCFAs) (i.e. acetate, propionate, butyrate, isobutyrate, valerate, and isovalerate), and d- and l-lactate.

FISH was applied to quantify the *Bifidobacterium* genus and *Eubacterium rectale*/*Clostridium coccoides* group (ER/CC) as described previously [18] using the 16S rRNA-targeted oligonucleotide probes S-G-Bif-0164 m-a-A-18 (5′-CATCCGGYATTACCACCC-3′) [34, 35] and S-*-Erec-0482-a-A-19 (5′-GCTTCTTAGTCARGTACCG-3′) [17], respectively.

### Data handling and statistical analyses

All analyses were performed on intention-to-treat data set (ITT), defined as all randomized subjects. Overall, the statistical analyses were performed comparing test with the control group per specimen analysed (saliva or faecal). The HBR group data was used as reference only and not as an intervention group. Statistical analyses were performed by using SAS^®^ (SAS Enterprise Guide version 4.3 or higher) for Windows (SAS Institute Inc., Cary, NC) unless indicated otherwise. Results are expressed and presented as mean values and standard deviations unless stated otherwise.

### 16S rRNA-gene sequencing data

The species diversity (α-diversity) indexes calculated in QIIME from the 16S rRNA-gene sequencing data were analysed at one single rarefied sequencing depth. The sequence depth which was selected for comparison was based on the maximum rarefaction depth where all or most of the samples were still included. Differences between treatment groups across time were tested using a random intercept mixed model including baseline in the outcome vector, adjustment for stratification factor (skin or gastrointestinal symptoms), treatment, time and treatment by time interaction as fixed factors. For assessing the treatment effect over time, significance of the treatment by time interaction was used.

The non-rarefied OTU tables obtained from QIIME were trimmed, removing sparse OTUs with at most 10 non-zero observations. Statistical analysis of the bacterial compositions was performed by applying a combination of multivariate analysis with Canoco 5 software [36], followed by differential abundance testing using the two-part statistics method [37]. Firstly, the constrained ordination method, known as redundancy analysis (RDA), was used to test time-dependent treatment effects with adjustment for stratification factor. The Monte Carlo permutation test (MCPT) with 1000 permutations was used to evaluate statistically significant differences (P ≤ 0.05) of the resulting model. If found significant, the top-15 responding OTUs identified from the RDA model were subsequently tested for differential abundance at the different timepoints using the two-part statistics method [37]. If the two-part statistics method could not be applied due to a small number of non-zero observations, then only presence-absence was considered by applying the Chi square test (if ≥ 5, but < 10 non-zeros in both groups) or Barnard test (if < 5 non-zeros in one group). The Benjamini–Hochberg false-discovery rate (FDR) was used to correct for multiple comparisons in the differential abundance tests [38] and significance was considered when FDR ≤ 0.1 at week 8 or when FDR ≤ 0.1 for at least two visits (i.e. 12 and 26 weeks).

### Other faecal parameters (pH, FISH outcomes, SCFAs, lactic acids)

The following rule was applied to faecal parameters that were subject to limit of detection (LOD): If a value was below detection limit and the percentage of values below detection limit was at most 30%, then the value was replaced with LOD/2. For parameters with more than 30% of the values below LOD only presence-absence was considered, and P-values were based on a logistic regression model. The P-values for continuous data were based on the analysis of covariance (ANCOVA) or Van Elteren test depending on normality of the residuals. All statistical models were corrected for baseline levels (if applicable) and stratification factor, and for statistical significance P ≤ 0.05 was considered.

## Results

**Fig. 1:**
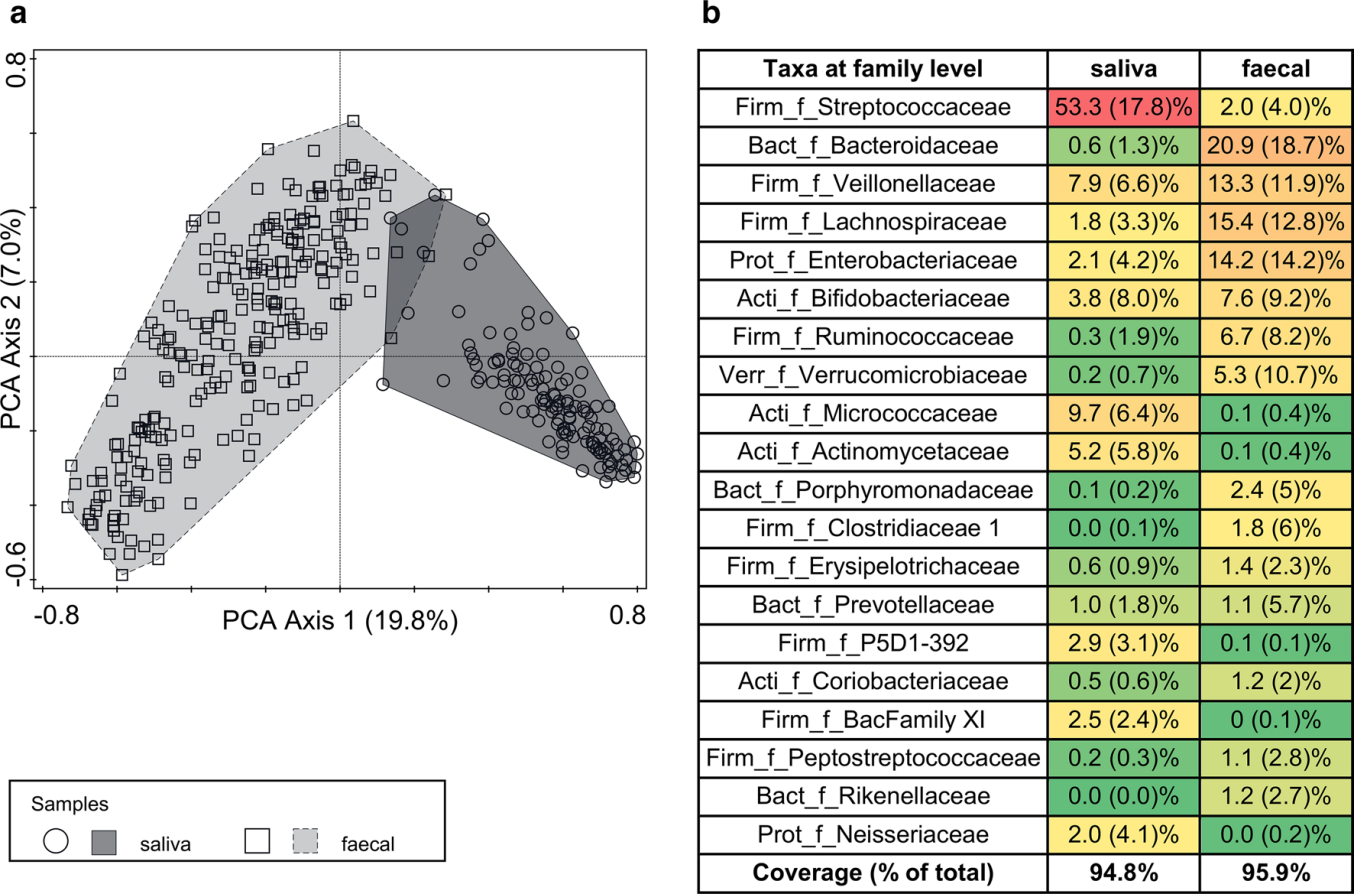
Principal component analysis (PCA) of faecal and salivary microbiota composition (**a**) and summary of major bacterial families identified (**b**). The PCA sample scatterplot is displayed on the first two axes summarizing most of the species variation, which is based on the OTU count data for each sample. The distance between the sample symbols (rounds for saliva and squares for faecal) approximates the dissimilarity of their species composition as measured by their Euclidean distance. Mean relative abundances (± SD) are summarized at the family level (“_f_”) for taxa > 1% and summarized in the heat map (red–yellow–green color scheme indicating high to low relative abundance). Abbreviations used for bacterial phylum levels: *Acti* actinobacteria, *Bact* bacteroidetes, *Firm* firmicutes, *Prot* proteobacteria, *Verr* verrucomicrobia

Subject characteristics were well balanced between groups as reported previously [15]. In total, 378 (125 saliva and 253 faecal) samples were successfully sequenced with a sequencing depth ranging from 15,265 to 129,780, and a median depth of 39,761 sequences per sample (Table 1). Principal component analysis (PCA), which is an unconstrained ordination method [36], was used to explore the taxonomic compositions of saliva and faecal samples. A clear clustering by sample origin was observed (Fig. 1a), which confirms that community composition is primarily determined by body habitat [39]. A summary of the most dominant taxa identified at the bacterial family level showed that saliva compared to faecal samples were typically characterized by increased relative abundance of *Streptococcaceae* (53.3 ± 17.8%), *Microcrococcaceae* (9.7 ± 6.4%) and *Actinomycetaceae* (5.2 ± 5.8%). Faecal samples were typically characterized by *Bacteroidaceae* (20.9 ± 18.7%)*, Lachnospiraceae* (15.4 ± 12.8%), *Enterobacteriaceae* (14.2 ± 14.2%), *Bifidobacteriaceae* (7.6 ± 9.2%), *Ruminococcaceae* (6.7 ± 8.2%) and *Verrucomicrobiaceae* (5.3 ± 10.7%) (Fig. 1b).

**Table 1:** Summary of saliva and faecal samples of subjects with CMA (ITT) and the healthy reference group (age-matched to week 8)

	Saliva samples		Faecal samples			Grand total
	Test (N = 35)	Control (N = 36)	Test (N = 35)	Control (N = 36)	Healthy subjects (N = 51)	
Week 0						
n subjects	35 (100%)	36 (100%)	29 (83%)	33 (92%)		
Mean (SD)	30,258 (14,753)	28,802 (6845)	47,476 (14,165)	47,269 (22,263)		
Week 8						
n subjects	24 (69%)	30 (83%)	24 (69%)	31 (86%)	48 (94%)	
Mean (SD)	27,387 (6555)	29,724 (15,773)	47,037 (13,765)	47,909 (16,532)	51,871 (19,134)	
Week 12						
n subjects			18 (51%)	26 (72%)		
Mean (SD)			52,921 (13,491)	51,391 (19,953)		
Week 26						
n subjects			21 (60%)	23 (64%)		
Mean (SD)			51,706 (22,464)	55,888 (17,966)		
Grand total	Saliva samples		Faecal samples			All samples
n samples	125		253			378
Mean (SD)	29,160 (11,837)		50,200 (18,201)			43,242 (19,124)

Sequence depth, after filtering for low quality reads, is given as mean (± SD)

### Bacterial diversity

**Fig. 2:**
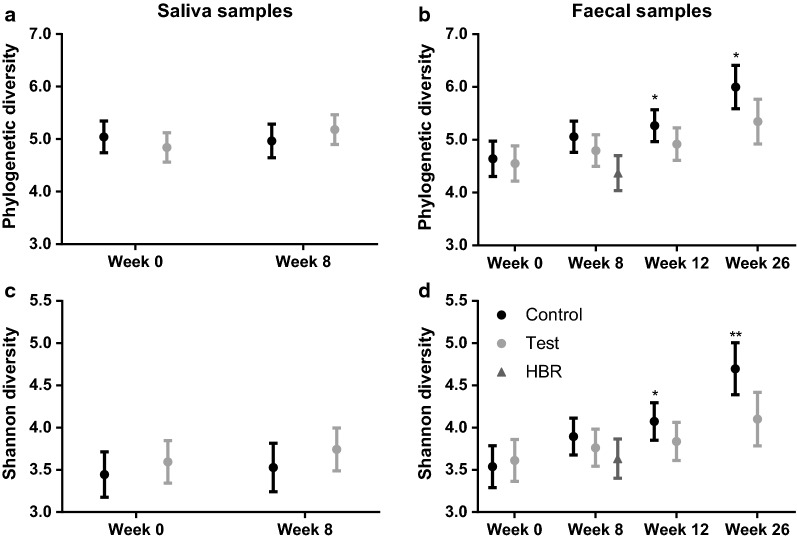
Plots summarizing the bacterial diversity of saliva (**a**, **c**) and faecal samples (**b**, **d**) using Faith’s phylogenetic diversity and the Shannon index, respectively. The symbols and error bars show the least square (LS) means with 95% CI for treatment by time. The faecal bacterial diversity of the HBR reference group (age-matched to week 8) is plotted as well. P-values are based on a random intercept mixed model with week 8/12/26 values as outcome, stratification factor and baseline values as covariate and treatment as fixed effect: *P ≤ 0.05 and **P ≤ 0.01

The species diversity indexes (PD and Shannon) were analysed at a rarefaction depth of 16,114 sequences per sample, which omitted one saliva sample (control, week 8) from comparison. Control and test group did not differ in salivary species diversity based on PD (Fig. 2a) or Shannon index (Fig. 2c). A treatment effect over time was observed for faecal species diversity, which was characterized by a more gradual increment (from baseline until 26w) in test compared to control for both PD (Fig. 2b, estimated difference per week = − 0.022, P = 0.069) and Shannon index (Fig. 2d, estimated difference per week = − 0.026, P = 0.005). The estimated average difference between test versus control was significantly different at week 12 (PD = − 0.349, P = 0.031 and Shannon = − 0.236, P = 0.049) and week 26 (PD = − 0.653, P = 0.012 and Shannon = − 0.596, P = 0.002). The HBR group showed the lowest average diversity (PD = 4.37 ± 1.14 and Shannon = 3.63 ± 0.80) compared to both test (PD = 4.89 ± 1.05 and Shannon = 3.75 ± 0.67) and control (PD = 5.17 ± 0.88 and Shannon = 4.01 ± 0.71) at week 8.

### Time-dependent treatment effects on oral microbiota

**Fig. 3:**
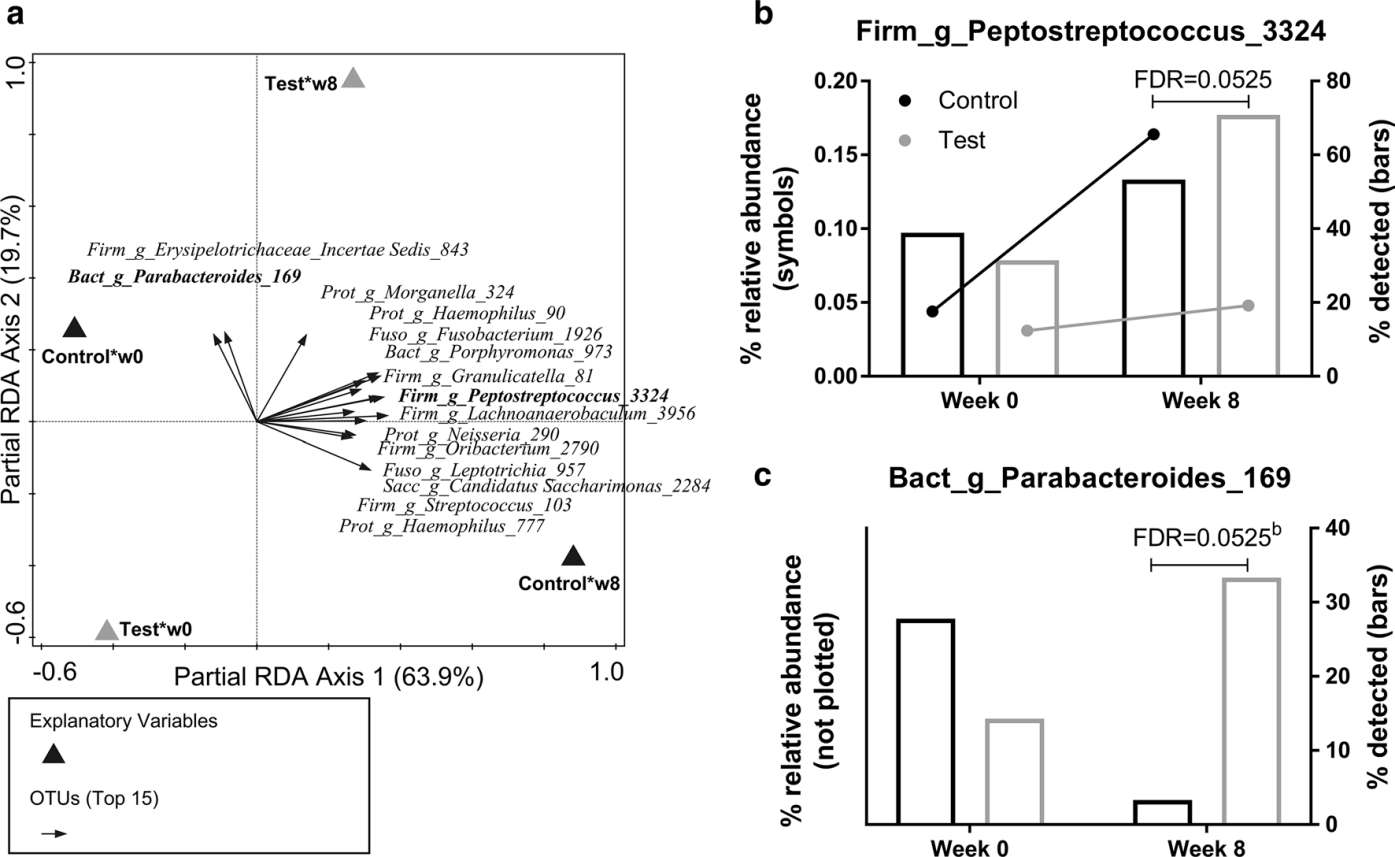
Redundancy analysis (RDA) biplot showing the change in species composition from week 0 to week 8 in saliva samples of infants receiving test or control formula (**a**). The top 15 OTUs are plotted based on best fit with the first two RDA axes. In bold the OTUs that were significantly different at week 8 between test versus control as confirmed with the two-part statistics method. The proportion of zeros (plotted as bars) and the median of the non-zero values (plotted as points) are displayed for the two differentially abundant OTU’s assigned to *Peptostreptococcus* (**b**) and *Parabacteroides* (**c**), respectively. False discovery rate (FDR) was used to correct the raw P-values for multiple testing with significance at 0.1. ^b^Only the Barnard test was performed (if < 5 non-zeros in one group) to compare the proportion of zeros. The OTU‘s are summarized with unique (but arbitrary) numbers as identifiers, and genus level (“_g_”) and phylum level taxonomic assignments: *Acti* actinobacteria, *Bact* bacteroidetes, *Firm* firmicutes, *Fuso* fusobacteria, *Prot* proteobacteria, *Sacc* saccharibacteria

Redundancy analyses (RDA) were carried out to test the effect of treatment (test/control) across time on the salivary community composition. We fitted both an RDA with and without correction for timepoint (baseline and week 8) and compared the results of the MCPT on the first axis of the model. The *P* value for the RDA with correction for timepoint (0.3816) was larger than our pre-set threshold of 0.05, so we used the simpler model (with P = 0.003) as a basis for interpreting the time-dependent treatment effects. The top 15 OTUs with the best fit on the first two axes (explaining most of the variation) were plotted in the RDA (Fig. 3a) and further evaluated for differential abundances between test and control using the two-part statistics method [37]. No differences were observed at baseline (based on FDR ≤ 0.1), but two OTUs out of this top 15 were found differentially abundant between test and control at week 8. This included a decreased relative abundance of *Peptostreptococcus* sp. (Fig. 3b, FDR = 0.0525) and an increased presence of *Parabacteroides* sp. (Fig. 3c, FDR = 0.0525).

### Time-dependent treatment effects on gut microbiota

**Fig. 4:**
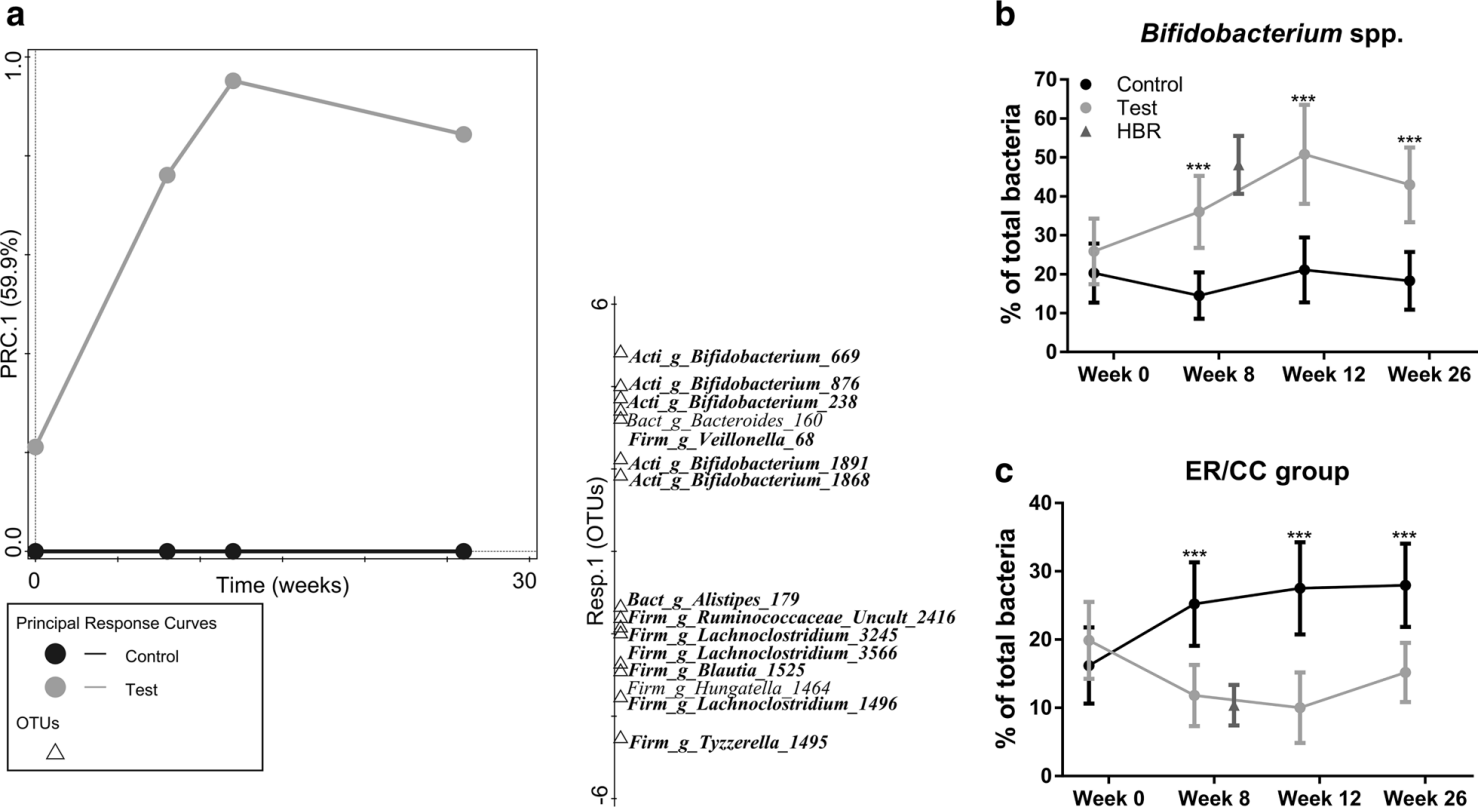
Principal response curves (PRC) of faecal microbiota composition showing the change in species composition of infants receiving test formula as compared to control (**a**). The diagram shows the first component of the PRC on the vertical axis and time on the horizontal axis with the treatments plotted as single response curves using control as reference with zero PRC values and so its curve lays over the horizontal axis. The top 15 OTUs are plotted on the separate vertical (one-dimensional) plot based on best fit with the first component of the PRC. In bold the OTUs that were significantly increased (> 0) or decreased (< 0) in Test versus Control at week 8 or at two or more timepoints (week 8/12/26) as confirmed by the two-part statistics (FDR ≤ 0.1). Percentages (means with 95% CI) of bifidobacteria (**b**), and ER/CC group (**c**) quantified by FISH at week 0/8/12/26. The HBR reference values (age-matched to week 8) are plotted as well. P-values are based on ANCOVA comparing Test versus Control with Week 8/12/26 values as outcome, stratification factor and baseline values as covariate and treatment as fixed effect: **P ≤ 0.01; ***P ≤ 0.001. Taxa names are given at the OTU level with unique (but arbitrary) numbers as identifiers, genus level (“_g_”), family level (“_f_”): *Bact* bacteroidaceae, *Bifi* bifidobacteriaceae, *Lach* lachnospiraceae, *Rumi* ruminococcaceae, *Veil* veillonellaceae; and phylum level: *Acti* actinobacteria, *Bact* bacteroidetes, *Firm* firmicutes

In order to assess the time-dependent treatment effects for the faecal community composition we used the Principal Response Curves (PRC) method [40]. The PRC is based on the RDA method, in which the principal component is plotted against time (baseline, week 8, 12 and 26) to enable the assessment and visualization of time-dependent treatment effects. The MCPT applied to test the significance of the resulting PRC model was significant for the first axis (P = 0.001). The top 15 OTUs with the best fit on the first axis were plotted (Fig. 4a) and further evaluated with the two-part statistics method [37]. No differences were observed at baseline, but a total of 13 OTUs out of the top 15 were confirmed to be differentially abundant between test and control at week 8 or at 2 or more timepoints. This included increased relative abundances in test versus control of 6 OTUs, of which 5 were assigned to *Bifidobacterium* and 1 was assigned to the *Veillonella* genus. The other 7 OTUs showed decreased relative abundances, of which 5 were assigned to 3 genera within the *Lachnospiraceae* family (*Tyzzerella*, *Blautia* and *Lachnoclostridium*) and 2 were assigned to the genera *Ruminococcus* and *Alistipes*, respectively.

### FISH quantification of faecal bacterial groups

The treatment effects on gut microbiota, as revealed by 16S rRNA-gene sequencing, were mostly associated with a relative increase of several species of the genus *Bifidobacterium* and a decrease of several species of the family *Lachnospiraceae*. FISH enumeration of these two bacterial groups was used to verify the absolute differences in abundance between treatments, of which results have been reported before [15, 16]. In summary, FISH analysis confirmed a significant enrichment of bifidobacteria in test versus control across time (Fig. 4b). Moreover, the proportions for test (36.0 ± 22.4%) as compared to that of the control group at week 8 (14.5 ± 16.4%) were close to the levels observed for the HBR group (48.1 ± 26.5%). The FISH probe used to quantify the ER/CC group targets the majority of *Lachnospiraceae* spp. including the differentially abundant OTUs associated with the genera *Tyzzerella*, *Blautia* and *Lachnoclostridium* as identified with 16S rRNA-gene sequencing. A decreased abundance of the ER/CC group in test versus control across time confirm these findings (Fig. 4c). Additionally, the levels for test (11.8 ± 10.9%) as compared to that of the control group at week 8 (25.2 ± 16.9%) were close to the levels observed for the HBR group (10.4 ± 10.6%).

### Faecal pH, SCFA and lactate

**Fig. 5:**
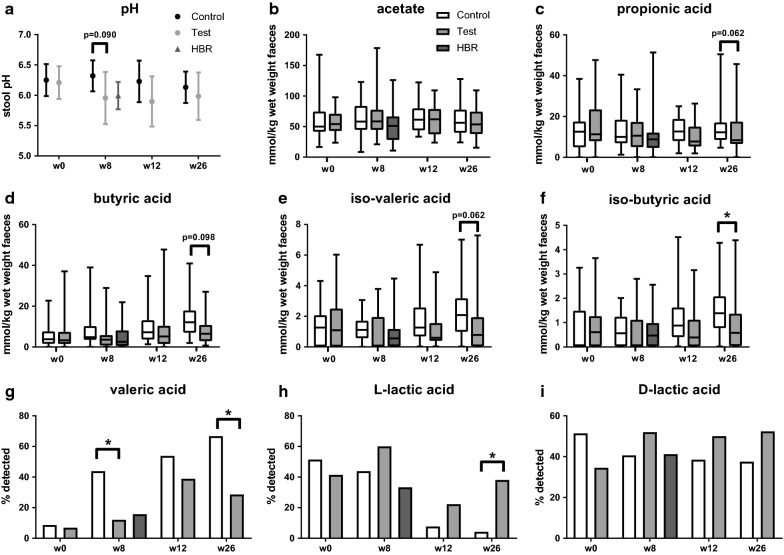
Mean with 95% CI of stool pH (**a**) for treatment by time. The box-plots summarize the amounts (in mmol/kg) of acetate (**b**), propionate (**c**), butyrate (**d**), iso-valerate (**e**), iso-butyrate (**f**) for treatment by time, respectively. Percentage of faecal samples (plotted as bars) with detectable levels of valerate (**g**), l-lactate (**h**), and d-lactate (**i**) for treatment by time, respectively. The HBR reference values (age-matched to week 8) are plotted as well. P-values for stool pH and acetate are based on ANCOVA comparing Test versus Control with week 8/12/26 values as outcome, stratification factor and baseline values as covariate and treatment as fixed effect. P-values for the variables summarized in **c**–**e** are based on Van Elteren test comparing test versus control with respect to change from baseline at week 8/12/26, taken the stratification factor into account. P-values for the variables summarized in **g**–**i** are obtained from a logistic regression model comparing test versus control at week 8/12/26 with adjustment for baseline values. *P ≤ 0.05

To assess whether the observed changes in gut microbiota composition also led to changes in gut physiology and microbial metabolites produced, the faecal pH and levels of SCFA and lactate were determined. No statistically significant differences were observed for faecal pH, acetate, propionate, butyrate, iso-valerate (Fig. 5a–e) and d-lactate (Fig. 5i) at the different timepoints. l-lactate was detected in a greater number of samples in test versus control at week 26 (38 vs. 4%, P = 0.020) (Fig. 5h). In contrast, valerate was detected in a smaller number of samples in test versus control at week 8 (44 vs. 12%, P = 0.036) and week 26 (67 vs. 29%, P = 0.021) (Fig. 5g). Moreover, the concentration of iso-butyrate was lower in test versus control at 26 weeks (P = 0.050) (Fig. 5f).

### Correlations of faecal microbiota composition and metabolic activity across time

**Fig. 6:**
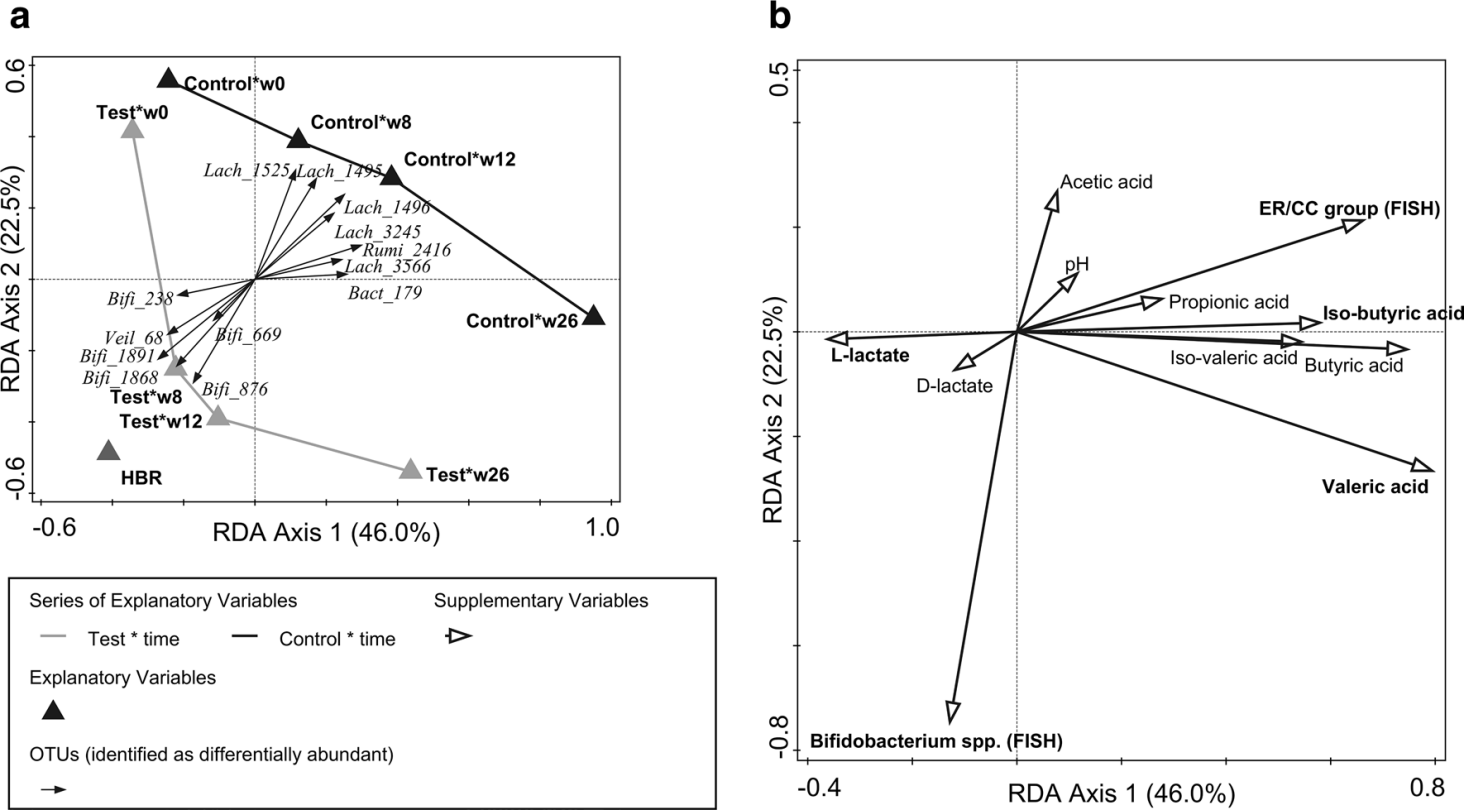
RDA plot of faecal microbiota composition based on treatment by time interaction and the HBR reference group (age-matched to week 8) as explanatory variables (**a**). OTUs that were identified as differentially abundant between test and control are plotted as biplot arrows in the RDA plot on the left. The same RDA plot is shown on the right (**b**), but then supplemented with faecal pH, FISH counts and levels of SCFA and lactic acids as correlation biplot. Variables that were significantly different between test and control are shown in bold. Taxa names are given at the OTU level with unique (but arbitrary) numbers as identifiers and the family level (“_f_”): *Bact* bacteroidaceae, *Bifi* bifidobacteriaceae, *Lach* lachnospiraceae, *Rumi* ruminococcaceae, *Veil* veillonellaceae

A redundancy analysis was used to summarize the faecal microbiota composition over time as explained by treatment (Test or Control) and the HBR group (Fig. 6a). The RDA recapitulates the results of the PRC analysis, but in addition confirmed the proximity in community composition of the test group at week 8 with the HBR. The additional faecal parameters measured (FISH, pH, SCFAs and lactic acids) were supplemented to this RDA in a separate biplot (Fig. 6b). An inverse correlation was observed for the FISH quantified levels of *Bifidobacterium* spp. with the ER/CC group, which reflects the major differences observed for test (and HBR) with the control group. Moreover, the increase of *Bifidobacterium* spp. in test was positively correlated with increased levels of l-lactate. In contrast, the more abundant levels of the ER/CC group across time in control was associated with increased levels of butyrate, valerate, iso-butyrate and iso-valerate. In test, the ER/CC group gradually increased from 12 to 26 weeks, which was associated (similarly as for control) with an increment of butyrate, valerate, iso-butyrate and iso-valerate at 26 weeks.

## Discussion

We previously reported the specific enhancement of bifidobacteria and decrement of the ER/CC group in the faeces of infants receiving the AAF with synbiotics consisting of a prebiotic blend of oligofructose and long-chain inulin and the probiotic strain *Bifidobacterium breve* M-16V [15, 16]. In this study, we applied a 16S rRNA-gene sequencing approach on both faecal and saliva specimens to elucidate more specifically which taxa responded to the intervention within the respective bacterial communities and what the effect was on their diversity and functionality.

We demonstrated that the effect of the synbiotic-containing AAF on infant microbiota was most pronounced for the gastro-intestinal tract and only minimally affected the oral microbiota. The AAF including synbiotics compared to the AAF without synbiotics showed a more gradual increment over time of bacterial diversity, which is also typically observed in longitudinal studies investigating early life gut microbiota development of breastfed infants as compared to formula-fed infants [7, 8, 41, 42]. These studies showed that the lower diversity of gut microbiota in breastfed infants is not only observed during the exclusive human milk-feeding period, but also during the complementary feeding-period until full transition to family foods, which reflects the sustained effects of human milk oligosaccharides on the bifidobacterial species that effectively thrive on these compounds [7, 8]. The AAF including synbiotics was found to enhance the bifidobacterial community, as several bifidobacterial species had increased, which was also reflected by an increase of the fermentation end-product l-lactate in the faeces of these infants. Interestingly, the concordant increase observed in this study for *Veillonella* sp. is most likely explained by the ability of this species to utilize and convert lactate into propionate [43]. In contrast, the infants receiving the control formula showed an early adoption of adult-like bacterial taxa belonging to the ER/CC group (resembling *Lachnospiraceae* spp.), namely *Tyzzerella*, *Blautia* and *Lachnoclostridium* spp., as well as species of *Ruminococcus* and *Alistipes*. This increase of adult-like taxa was associated with an increase of valerate and the branched-chain SCFA iso-butyrate, which are fermentation products that result from the degradation of proteins and amino acids [44, 45]. Overall, these results indicate that the synbiotic-supplemented AAF induced a saccharolytic fermentation profile, while infants receiving the AAF without synbiotics showed a more proteolytic fermentation activity, which is generally associated with metabolite profiles that may be less beneficial for colonic health [46, 47].

To date, several case–control studies have specifically investigated the gastrointestinal microbiota of infants and children with confirmed CMA compared to age-matched healthy controls [48–52]. All of them reported altered gut microbiota in infants and children with CMA, although with mixed findings. However, the common characteristics that were identified in these studies included lower levels of bifidobacteria [49–52] and increased levels of members of the heterogenous ER/CC group [48–50]. In analogy with our study, the case–control study of Thompson-Chagoyan et al. [48] in addition observed increased fecal butyrate and branched-chain SCFA (iso-butyrate, iso-valerate) concentrations in CMA infants compared to healthy infants. Interestingly, our study demonstrated that 8-weeks use of the synbiotic-supplemented AAF approximated the composition and activity of the gut microbiota of the age-matched healthy, breastfed reference group.

Our study has several limitations as addressed before [15], which includes the challenges in making and confirming a specific and accurate diagnosis of non-IgE mediated allergy. The chance of including infants with other (food) allergy presentations were mitigated by applying a robust diagnostic work-up [15]. For a number of subjects, no specimens were available due to insufficient material or not completing the study until 26 weeks (Table 1), which limited the number of evaluable samples at week 12 and 26. This limitation was however similar in test and control groups and would, therefore, not have affected the observed differences between groups. Moreover, the identified microbial signatures showed very consistent patterns across time and were, regarding the relative abundances of bifidobacteria and the ER/CC group, independently confirmed by 16S rRNA-gene sequencing and FISH. Although, we specifically studied subjects with non-IgE-mediated CMA, Burks et al. [14] showed that an AAF, including ingredients from the current synbiotic blend, was safe in patients with IgE and non-IgE-mediated CMA, and affected the microbial signatures in an equal manner. Importantly, this study was primarily designed to investigate the effects of the synbiotic-containing AAF on gut microbiota and the suitability for the use in dietary management of CMA. As reported previously [14–16], the AAF including synbiotics showed reduction of allergic symptoms as seen for the control AAF, and in addition showed potential beneficial systemic effects based on the adverse events, which reported fewer subjects in test with infections and need for anti-infective medication, including antibiotics [14–16] and lower use of agents for dermatological purposes [16]. Currently investigations are ongoing to assess whether the AAF including synbiotics influences cow’s milk tolerance acquisition in a clinical trial conducted in infants with confirmed IgE-mediated CMA (Netherlands Trial Register NTR3725).

## Conclusions

Based on the data showing pronounced effects on gut microbiota composition, diversity and metabolic activity, we conclude that the AAF including the specific synbiotics offers an effective nutritional strategy to modulate the gut microbiota of infants with suspected non-IgE-mediated CMA closer to a healthy breastfed profile.

## Data Availability

The data that support the findings of this study are available from Nutricia Reseach, but restrictions apply to the availability of these data, which were used under license for the current study, and so are not publicly available. Data are however available from the corresponding author upon reasonable request and with permission of Nutricia Research and respecting the EU GDPR regulation.

## Abbreviations

16S rRNA: 16S ribosomal RNA
AAF: amino-acid based formulas
ANCOVA: analysis of covariance
CMA: cow’s milk allergy
eHF: extensively hydrolysed formula
ER/CC: *Eubacterium rectale*/*Clostridium coccoides* group
FDR: false-discovery rate
FISH: fluorescent in situ hybridization
HBR: healthy, breastfed infants reference group
IgE: immunoglobulin E
ITT: intention-to-treat
LOD: limit of detection
MCPT: Monte Carlo permutation test
OTUs: operational taxonomic units
PCA: principal component analysis
PD: phylogenetic diversity
PRC: principal response curves
QIIME: quantitative insights into microbial ecology
RDA: redundancy analysis
SCFAs: short-chain fatty acids
SD: standard deviation
